# Mitoquinone Prevents Cardiac Dysfunction by Normalizing Mitochondrial ROS and Calcium Handling in Acute Myocardial Infarction

**DOI:** 10.1111/apha.70276

**Published:** 2026-07-10

**Authors:** Carolina Falcão Ximenes, Pietra Zava Lorencini, Carmen Castardeli, Marlon Ramos Rosado Machado, Katyana Kaline Silva Ferreira, Sara Bianca Oliveira Mendes, Anna Karolina Nascimento Costa, Mario Morais Silva, Sérgio Ricardo Aluotto Scalzo, Marcos Eliezeck, Silvia Guatimosim, Eduardo Hertel Ribeiro, Donald M. Bers, Aurélia Araujo Fernandes, Ivanita Stefanon

**Affiliations:** ^1^ Post‐Graduate Program in Physiological Sciences Federal University of Espírito Santo Vitória Espírito Santo Brazil; ^2^ Department of Physiology and Biophysics Federal University of Minas Gerais Belo Horizonte Minas Gerais Brazil; ^3^ Department of Pharmacology University of California Davis Davis California USA; ^4^ Department of Morphology Federal University of Espírito Santo Vitória Espírito Santo Brazil

**Keywords:** mitochondria, myocardial contractility, myocardial infarction, oxidative stress, reactivity oxygen species

## Abstract

Acute myocardial infarction (MI) is the leading cause of heart failure (HF). However, the role of mitochondrial ROS (ROSm) in early MI dysfunction remains unclear. This study aimed to evaluate the impact of MitoQ on cardiac function in cases of heart HF following MI. Male Wistar rats were divided into four experimental groups: Sham, Infarct, Sham+MitoQ, and Infarct+MitoQ. MitoQ was administered orally (8 mg/kg/dia) for 7 days. Hemodynamic parameters, infarct area, papillary muscle contractility, cardiomyocyte mechanics, Ca^2+^ transients, and total and mitochondrial superoxide (DHE and MitoSOX) were assessed. After 7 days of MI, rats exhibited impaired contractility, altered inotropic response to extracellular Ca^2+^, cardiomyocyte hypertrophy, and increased total and ROSm. MitoQ prevented body weight loss and significantly improved hemodynamic parameters compared to the Infarct group. In papillary muscles, MitoQ restored basal isometric force and the inotropic response to extracellular Ca^2+^. In cardiomyocytes, it attenuated hypertrophy, preserved shortening, and reduced ([Ca^2+^]i) transient amplitude. MitoQ significantly decreased total and mitochondrial O_2_•^−^ production. It selectively reduced NOX1 expression under simulated conditions but did not significantly affect NOX2, SOD1, or catalase expression in the context of MI. MitoQ prevented contractile dysfunction, suggesting that mitochondrial oxidative stress plays a decisive role in myocardial dysfunction during the acute phase of MI. Targeting antioxidant therapy to the mitochondria represents a promising strategy for preventing post‐infarction heart failure and opens new perspectives for the development of more effective interventions in the treatment of cardiovascular diseases.

## 1 Introduction

Myocardial infarction (MI) remains a leading cause of morbidity and mortality worldwide. This acute event triggers a cascade of hemodynamic, inflammatory, and metabolic responses that frequently culminate in adverse ventricular remodeling and cardiac dysfunction [1, 2]. A central feature of MI is the increased oxidative stress resulting from excessive production of reactive oxygen species (ROS) and an overwhelmed antioxidant defense system [3, 4, 5]. Mitochondrial dysfunction plays a pivotal role in this process, as mitochondria are both a primary source and target of ROS, exacerbating cellular dysfunction and driving the progression of cardiac injury [6, 7].

Studies have identified mitochondrial‐derived ROS (mROS) as critical mediators of calcium dysregulation, apoptotic signaling, and fibrotic pathways in the post‐MI heart. Specifically, mROS disrupt intracellular calcium homeostasis by oxidizing calcium‐handling proteins such as the ryanodine receptor [8, 9] (RyR2) and sarcoplasmic/endoplasmic reticulum calcium ATPase (SERCA2a) [10]. This disruption promotes calcium leak from the sarcoplasmic reticulum (SR) and cytosolic calcium overload, leading to contractile dysfunction and maladaptive remodeling [11, 12, 13]. Moreover, evidence suggests a positive‐feedback loop between mROS and NADPH oxidases (NOX), particularly NOX2 and NOX4 isoforms, which amplifies oxidative stress and myocardial damage in the post‐MI setting [14, 15, 16].

Pharmacological interventions targeting mROS have shown significant therapeutic promise. Mitoquinone mesylate (MitoQ), a mitochondria‐targeted antioxidant, has demonstrated efficacy in reducing oxidative stress and improving cardiac function in hypertrophy and heart failure [17, 18]. However, its effects during the acute phase of MI, particularly its influence on contractile function and the interplay between mROS and calcium homeostasis, remain poorly understood.

This study aims to evaluate whether MitoQ administration can prevent contractile dysfunction and ventricular remodeling during the early post‐MI period. We hypothesize that MitoQ‐mediated reduction of mROS attenuates hemodynamic alterations, improves intracellular calcium homeostasis, and preserves contractile function.

## 2 Materials and Methods

### 2.1 Experimental Groups

All experimental protocols adhered to the National Institutes of Health (NIH) guidelines and were approved by the local animal ethics committee (protocol #16/2021, CEUA‐UFES) at the University of Espírito Santo.

In brief, rats were anesthetized with ketamine (50 mg·kg^−1^) and xylazine (10 mg·kg^−1^) before undergoing a left lateral thoracotomy between the fourth and fifth intercostal spaces. Upon exteriorization of the heart, the left atrium was gently retracted to facilitate ligation of the left coronary artery with a 6–0 mononylon suture, placed between the pulmonary artery's exit point and the left atrium. The heart was then repositioned within the thoracic cavity, and the incision was closed using 1–0 cotton sutures. This procedure was performed to induce transmural infarction across 40%–60% of the left ventricular surface while avoiding damage to the interventricular septum.

Following surgery, animals were allocated into four groups: Sham, Sham + MitoQ, Myocardial Infarction (MI), and MI + MitoQ. Rats in the MitoQ treatment groups received MitoQ (Mitoquinone Mesylate 100 μM; corresponding to 8 mg/kg/day) in their drinking water for a period of 7 days. Although MitoQ is commercially available, the compound used in this study was generously provided by Dr. Mike Murphy. All rats had *ad libitum* access to tap water and standard laboratory chow.

### 2.2 Hemodynamic Measurements

Seven days after the induction of MI, rats were anesthetized with ketamine (50 mg·kg^−1^) and xylazine (10 mg·kg^−1^), intraperitoneally. The left carotid artery was carefully isolated to prevent nerve damage. A tapered polyethylene cannula (PE‐50), prefilled with heparinized saline (100 U/mL), was gently inserted into the left carotid artery. This setup enabled continuous monitoring of blood pressure (BP) and heart rate (HR) and allowed further insertion into the left ventricle for assessing left ventricular systolic pressure (LVSP) and end‐diastolic pressure (LVEDP), as well as for measuring the rate of pressure change (dP/dt_max and dP/dt_min). Real‐time data acquisition was achieved using a TSD 104A pressure transducer connected to an MP100 amplifier and data acquisition system (Biopac Systems Inc., CA, USA), facilitating comprehensive analysis of cardiac function.

### 2.3 Infarct Size Measurement

To register cardiac electrical activity, animals were placed on the surgical table and electrocardiographic recordings were taken. A preamplifier (BioAmp, AdInstrumens, Australia) connected to a system of data acquisition (PowerLab, AdInstruments, Australia) was used to record the ECG parameters. Electrodes were placed in lead I configuration to record the ECG and confirm the occurrence of MI, identified by the presence of persistent ST‐segment elevation and the development of pathological Q waves, which are established criteria for experimental MI diagnosis.

The quantification of infarct size followed a previously described method [19]. Under a dissection microscope, the infarcted tissue was carefully separated from the viable cardiac tissue. Both the healthy and infarcted regions were outlined on graph paper, and the areas were measured to determine the infarct size. This size was expressed as a percentage of the total left ventricular area, including the septum.

### 2.4 Isolation of Papillary Muscle

The preparation of isolated left ventricular papillary muscles under isometric contraction conditions was conducted according to established protocols [19, 20]. Following hemodynamic measurements, rats received an intraperitoneal injection of 500 units of heparin. After a 10‐min interval, the hearts were rapidly excised and perfused through the aortic stump to facilitate dissection of the posterior left ventricular papillary muscle.

The isolated papillary muscles were mounted for isometric tension recording in 25 mL of Krebs–Henseleit solution (composition in mM: 135 NaCl; 4.6 KCl; 1.25 CaCl2; 1.15 MgSO4; 1.2 KH2PO4; 11 glucose; pH = 7.4) at 30°C, continuously aerated with a gas mixture of 95% O_2_ and 5% CO_2_. Twitch contraction was controlled by delivering isolated rectangular pulses (10 V, 12 ms duration) through platinum electrodes. The standard stimulation rate was set to 0.5 Hz to maintain steady‐state contractions. Resting tension was adjusted to achieve maximal contractile force (L_max). Throughout the experiment, the posterior papillary muscle was secured with a set of rings, with one end fixed and the other connected to a force transducer (TSD125, Biopac Systems Inc., CA) linked to an amplifier (DA100C, Biopac Systems Inc., CA), all within a glass container containing 25 mL of the nutritive solution. Isometric force development was normalized to muscle weight (g/mg). Recordings commenced after a 60‐min adaptation period, allowing the muscle to acclimate to the experimental conditions.

The following protocols were subsequently applied: first, twitch contractions were recorded under steady‐state conditions. To assess SR activity, pause intervals of 15, 30, and 60 s were introduced. SR activity was evaluated via post‐rest potentiation, where the amplitude of the first contraction after a pause (post‐rest contraction) was compared to steady‐state contractions. To reduce variability from differing contraction amplitudes, results are presented as relative potentiation (the ratio of post‐rest contraction amplitude to steady‐state contraction amplitude).

Next, the impact of varying extracellular calcium concentrations (0.62, 1.25, 2.5 and 3.75 mM) on force development was assessed. Lastly, the positive inotropic effect of cumulative concentrations of isoproterenol (10^−7^ to 10^−2^ M) was analyzed under steady‐state conditions (with CaCl_2_ at 0.62 mM).

### 2.5 Cardiomyocyte Isolation

The hearts were rapidly excised and perfused using the Langendorff method with Ca^2+^‐free modified Tyrode solution containing (in mmol/L): 130 NaCl; 5.4 KCl; 25 HEPES; 0.5 MgCl_2_; 0.33 NaH_2_PO_4_; 22 glucose and 100 U/mL insulin; pH = 7.4 until complete removal of blood was achieved. The hearts were then perfused with Tyrode solution containing 50 μmol CaCl_2_ and 1 mg/mL collagenase (Type II; Worthington, Lakewood, NJ) until they became softened (~15 min). Subsequently, the hearts were removed from the perfusion apparatus, minced into ~1‐mm pieces, and stirred for 4 min in Tyrode solution containing 50 μM CaCl_2_, 0.7 mg/mL collagenase, and 0.02 mg/mL protease. The cell suspension was filtered through a 200‐μm mesh to eliminate tissue debris, and the extracellular Ca^2+^ concentration was gradually increased to 1.8 mM over a 10‐min period through three centrifugation cycles. Experiments were conducted at room temperature (22°C–24°C).

For contractility analysis, the isolated cardiomyocytes were electrically stimulated by platinum electrodes (1 Hz, 30 V) with a pulse duration of 5 ms. A high‐speed digital CMOS camera (SILICON VIDEO 643 M, EPIX Inc.) was used to capture cell images at 200 fps, with a resolution of 640 × 480 pixels, a pixel size of 0.25 μm/pixel, and an 8‐bit depth. Cellular contraction and relaxation images were collected from the baseline state of each cell. Data on cellular contractility were obtained according to a previously described protocol [21]. To acquire cell contractility parameters, images were processed and analyzed using the CONTRACTIONWAVE software [21, 22]. The software automatically detected parameters including Maximum Contraction Speed (MCS), Maximum Relaxation Speed (MRS), percentage cell length shortening (Shortening Area), Contraction‐Relaxation Time (CRT), Contraction Time (CT), and Relaxation Time (RT).

For Ca^2+^ transient analysis, intracellular Ca^2+^ imaging experiments were conducted on cardiomyocytes loaded with 6 μM Fluo‐4 AM (Invitrogen, Eugene, OR) for 30 min, followed by a Tyrode solution wash containing 1.8 mM Ca^2+^ for the same period. The cardiomyocytes were paced at 1.0 Hz, and Ca^2+^ levels were expressed as F/F₀, where F₀ represents the baseline Ca^2+^ fluorescence. The T₅₀ parameter, representing the time from the peak Ca^2+^ transient to 50% decay, was calculated from F/F₀ fluorescence traces after digital image processing with ImageJ software.

Mitochondrial ROS were assayed in a separate group of cells that were incubated with 7.5 μM MitoSOX Red (MitoSOX, Molecular Probes, Eugene, OR, USA) for 10 min at 37°C, followed by washing with Tyrode solution containing 1.8 mM Ca^2+^ to remove excess dye. Imaging was performed using confocal line‐scan imaging on a Zeiss LSM 800 confocal microscope (CAPI Center/UFMG) equipped with a 63× oil‐immersion objective. MitoSOX was excited at 510 nm and emission was collected at 580 ± 20 nm. Line‐scan acquisition was performed at 512 pixels/line with a scan speed of 1.5 ms/line, and all images were acquired under identical laser power, pinhole, and detector gain settings to allow quantitative comparison between groups.

Cardiomyocyte surface area was measured in isolated ventricular myocytes fixed with 4% paraformaldehyde (PFA, pH 7.4, adjusted with NaOH) for 15 min at room temperature. After fixation, cells were washed three times with cold PBS. Images were acquired using a Zeiss LSM 880 confocal system. All images represent four independent experiments in which multiple cells were evaluated.

### 2.6 Western Blot Analysis

Proteins were separated on 7.5%, 10%, or 12% SDS‐PAGE gels, depending on the molecular weight of the target proteins, and subsequently transferred electrophoretically onto PVDF membranes (Cytiva, Wilmington, USA). Protein concentration was determined using the Lowry method, with bovine serum albumin (BSA) as the standard. To maximize membrane utilization and enable the detection of multiple targets, the membrane was cut into segments after the protein transfer. This approach was chosen because the targets had distinct molecular weights but similar protein quantities.

Each segment was separately incubated with monoclonal antibodies specific for the following proteins: NOX1 (50 μg, 1:500, Sigma SAB2108601), NOX2 (40 μg, 1:1000, Abcam ab129068), NOX4 (50 μg, 1:500, Sigma SAB1304615), SOD1 (50 μg, 1:500, Santa Cruz sc‐271 014), SOD2 (50 μg, 1:500, Santa Cruz sc‐133 134), Catalase (50 μg, 1:500, Sigma C0979), Serca2a (30 μg, 1:1000, Santa Cruz sc‐376 235), PLN (30 μg, 1:2000, Santa Cruz sc‐393 990), pPLN Ser16 (30 μg, 1:1000, Santa Cruz sc‐24 519) and pPLN Thr17 (30 μg, 1:1000, Santa Cruz sc‐24 565).

After incubation, the membrane segments were washed and treated with horseradish peroxidase (HRP)‐conjugated secondary antibodies: anti‐mouse IgG (A2554, Sigma, 1:15000) or anti‐rabbit IgG (A0545, Sigma, 1:15000). Immunocomplex detection was performed using an enhanced horseradish peroxidase/luminol chemiluminescence system (Pierce ECL, Thermo Scientific), and signals were captured using a Chemidoc imaging system (Bio‐Rad, USA).

Normalization was performed by total protein quantification using Stain‐Free imaging (Bio‐Rad). Quantitative analysis was conducted with ImageLab software (Bio‐Rad) by calculating the ratio of the target protein signal to the control.

### 2.7 In Situ Detection of ROS Production

The oxidative fluorescent dye dihydroethidium (DHE) was used to assess ROS production in situ. DHE permeates cell membranes and, upon oxidation by ROS, is converted to ethidium bromide, which intercalates with DNA and becomes trapped within the cell. Ethidium bromide is excited at 546 nm and emits fluorescence at 610 nm. Frozen left ventricular tissue samples were sectioned into 10‐μm‐thick slices and placed on glass slides. Serial sections were equilibrated under identical conditions for 30 min at 37°C in Krebs–HEPES buffer (composition in mM: 130 NaCl, 5.6 KCl, 2 CaCl_2_, 0.24 MgCl_2_, 8.3 HEPES, and 11 glucose, pH 7.4). Fresh buffer containing 2 μM DHE was applied to each section, which was then covered with a coverslip, incubated for 30 min in a light‐protected, humidified chamber at 37°C, and examined with an inverted fluorescence microscope (NIKON Eclipse Ti‐S, ×40 objective). Fluorescence was detected using a 568‐nm long‐pass filter, and mean fluorescence densities in the target regions were calculated. Tiron (1 mM) was used as a negative control.

### 2.8 Histological Analysis of Cardiac Tissue

Tissue samples were stored in 4% paraformaldehyde on the day of organ collection. After 72 h, the samples were transferred to 70% ethanol for fixation for a minimum period of 48 h. Subsequently, they were processed, embedded in paraffin at 58°C, and blocked for sectioning. For histological analysis of the heart, transverse sections of the cardiac tissue were obtained. Using a micrometer, the paraffin blocks were sectioned at a thickness of 5 μm and mounted on gelatin‐coated slides, which were stored until staining. Interstitial collagen analysis was performed using the Picrosirius Red stain.

The slides were deparaffinized in three consecutive baths containing only xylene, followed by one bath containing equal parts of xylene and ethanol. They were then hydrated in three successive baths of ethanol—two at 100% and one at 96%. Each bath lasted 3 min. The slides were washed with distilled water and stained with a Picrosirius Red solution for 5 min. They were washed again with distilled water and counterstained with eosin for 1 min. Finally, according to the post‐staining protocol, slides were dehydrated in reverse order of deparaffinization (three ethanol baths for 1 min each—one at 96% and two at 100%—followed by one bath of ethanol/xylene for 3 min and three baths of xylene for 3 min each) and mounted with coverslips.

Tissues were examined in multiple sections throughout the organ using an Olympus microscope at a final magnification of 100× (AX70; Center Valley, PA; up to 50 μm between sections). All images were captured using an AxioCam high‐resolution camera (2048 × 1536 pixels) and processed with AxioVision software (version 4.8).

### 2.9 Statistical Analyses

Results are presented as mean ± standard error of the mean (SEM). A two‐way analysis of variance (ANOVA) was conducted to evaluate potential changes induced by the factors of infarction and MitoQ treatment, both individually and interactively. Fisher's least significant difference (LSD) post hoc test was applied for multiple comparisons when significant differences were identified by ANOVA. An alpha level of 0.05 (*p* < 0.05) was considered statistically significant. The number of animals (*n*) used in each experimental protocol is indicated in parentheses. Protein expression data are presented as the ratio of the target protein signal to the loading control signal (total protein by stain‐free on the immunoblot).

## 3 Results

### 3.1 Global Parameters

**FIGURE 1:**
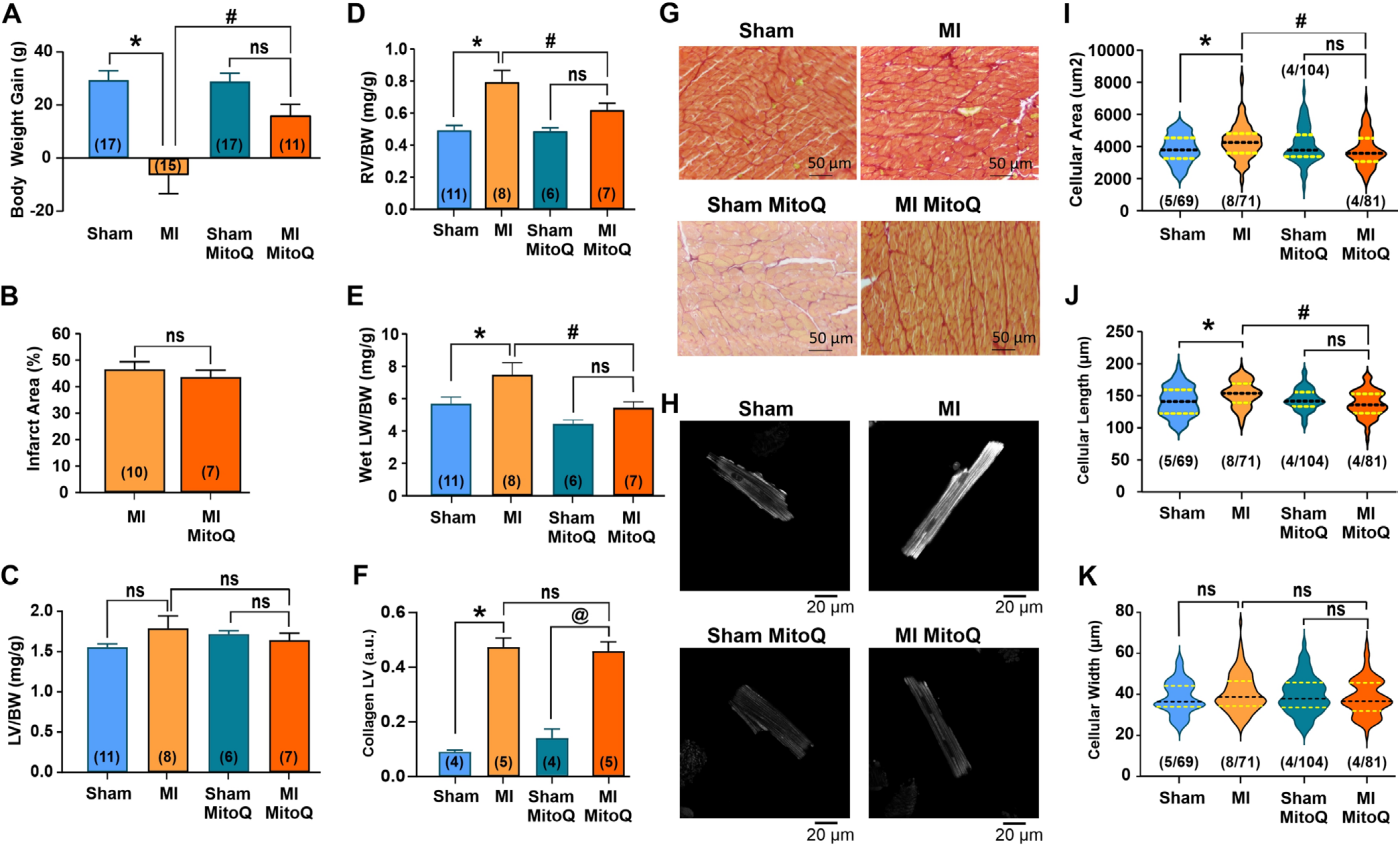
Effects of MitoQ on physiological and cellular parameters in a 7‐day myocardial infarction (MI) model. The following groups were analyzed: Sham, Sham MitoQ, MI, and MI MitoQ. (A) Body weight gain; (B) infarct size; (C) left ventricle‐to‐body weight ratio (LV/BW); (D) right ventricle‐to‐body weight ratio (RV/BW); (E) lung wet weight‐to‐body weight ratio (LW/BW); (F) collagen content in the left ventricle (LV); (G) interstitial collagen analysis in the LV assessed by Picrosirius Red staining; Scale = 50 μm; (H) representative images of isolated cardiomyocytes, Scale = 20 μm; (I) cell area; (J) cell length; (K) cell width. Data are presented as mean ± SEM. Statistical analysis was performed using two‐way ANOVA. *p < 0.05 vs. Sham; #*p* < 0.05 vs. MI; ^@^ *p* < 0.05 vs. Sham MitoQ; ns, not significant. The number of animals per group is indicated in parentheses.

The study aimed to evaluate the effects of the mitochondrial‐targeted antioxidant MitoQ on various physiological and cellular parameters in a MI rat model. Control animals subjected to MI experienced a significant reduction in body weight gain over the 7‐day observation period. In contrast, MitoQ treatment mitigated this weight loss, resulting in a notable increase in body weight gain in the MitoQ‐treated MI group compared to the untreated MI group (*p* < 0.05). This result indicates that MitoQ may have a protective role against the catabolic effects typically associated with MI (Figure 1A).

The infarct area was similar between groups (Figure 1B). Further analysis revealed a trend toward an increased left ventricle‐to‐body weight (LV/BW) ratio in the untreated MI group compared to controls, though this trend did not reach statistical significance (*p* = 0.06) (Figure 1C). However, the right ventricle‐to‐body weight (RV/BW) ratio was significantly elevated in the MI group, indicative of right ventricular hypertrophy as an adaptive response to cardiac injury. Remarkably, MitoQ treatment normalized the RV/BW ratio (*p* < 0.05 vs. MI), suggesting its ability to prevent pathological hypertrophy in the right ventricle (Figure 1D).

Pulmonary congestion, reflected by an increased lung‐to‐body weight ratio (Wet LV/BW), was observed in MI animals, a consequence of elevated pulmonary pressure secondary to cardiac dysfunction (Figure 1E). MitoQ treatment significantly reduced lung weight in MI animals (*p* < 0.05 vs. MI), indicating a potential benefit in reducing pulmonary congestion, which may reflect improved cardiac function.

Myocardial fibrosis, assessed by collagen content in the left ventricle, was significantly higher in the untreated MI group compared to controls, highlighting the structural remodeling that occurs following infarction (Figure 1F,G). However, MitoQ treatment did not reduce collagen deposition (*p* > 0.05), suggesting that it does not exhibit anti‐fibrotic effects, at least in the early stages following MI.

Cellular morphology analyses further demonstrated significant changes in cardiomyocytes from the MI group. The cells exhibited increased cellular area and length compared to those in the sham group (Figure 1H–J), reflecting hypertrophic remodeling in response to MI. MitoQ administration effectively prevented these morphological changes, as evidenced by reduced cell area and length (*p* < 0.05 vs. MI), indicating its potential to attenuate the hypertrophic response (Figure 1H–J). Cell width was unaffected between groups (Figure 1K).

### 3.2 Hemodynamics and Myocardial Contractility

**FIGURE 2:**
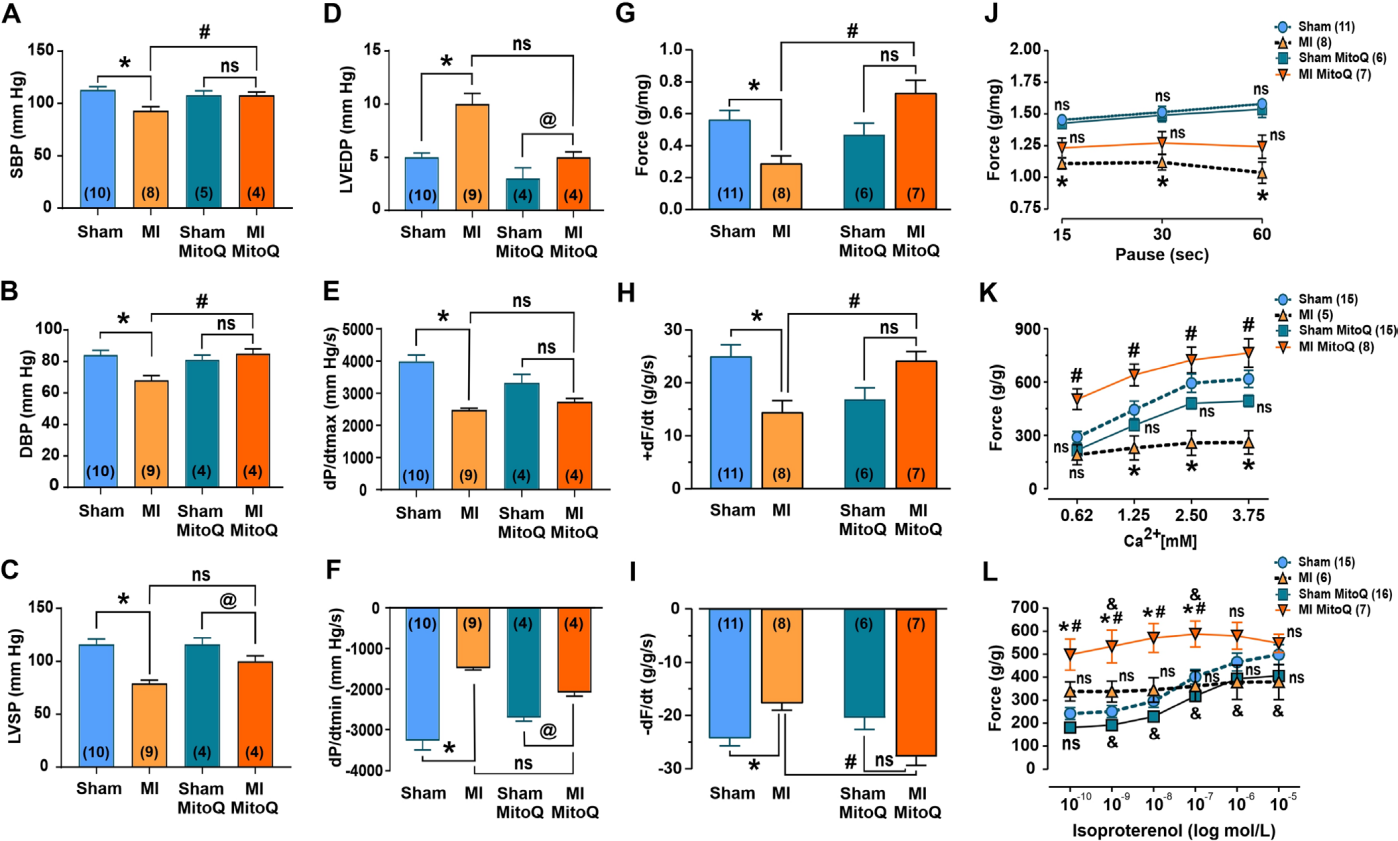
Impact of MitoQ treatment on hemodynamic and contractile parameters in rats following myocardial infarction (MI). (A) Both systolic pressure (SBP); (B) diastolic blood pressure (DBP); (C) left ventricular systolic pressure (LVSP); (D) left ventricular end‐diastolic pressure (LVEDP); (E) maximum rates of pressure development (+dP/dt_max); (F) minimum rates of pressure development; (G) isometric force (g/g) in the left ventricular papillary muscles at baseline (CaCl_2_ 1.25 mM); (H) positive first‐time derivative (dF/dt+); (I) negative first‐time derivative (dF/dt‐); (J) post‐rest potentiation (15, 30, and 60 s) (g/mg); (K) isometric force (g/g) in the left ventricular papillary muscles measured with different extracellular CaCl_2_ concentrations (0.62, 1.25, 2.5, and 3.75 mM). (L) Isometric force (g/g) in the left ventricular papillary muscles measured in response to different isoproterenol concentrations (10^−7^ to 10^−2^ M) in 0.62 mM calcium. Data are presented as mean ± SEM. Statistical analysis was performed using two‐way ANOVA. *p < 0.05 vs. Sham; #*p* < 0.05 vs. MI; ^@^p < 0.05 vs. Sham MitoQ; ns not significant; &*p* < 0.05 vs. 10^−10^M. The number of animals per group is indicated in parentheses.

The results in Figure 2 illustrate the impact of MitoQ treatment on hemodynamic and contractile parameters in rats following myocardial infarction (MI). MI caused a significant reduction in both systolic (SBP) and diastolic blood pressure (DBP) compared to the sham group (Figure 2A,B, *p* < 0.05), reflecting the diminished cardiac function that follows infarction. Notably, MitoQ treatment restored both SBP and DBP to levels comparable to those in the sham group, indicating a protective role of MitoQ in maintaining blood pressure post‐MI (Figure 2A,B, *p* < 0.05).

In terms of left ventricular function, MI led to a significant reduction in left ventricular systolic pressure (LVSP) and an increase in left ventricular end‐diastolic pressure (LVEDP), indicative of systolic and diastolic dysfunction, respectively (Figure 2C,D). MitoQ treatment prevented these changes, preserving LVSP and reducing LVEDP compared to the untreated MI group. This finding suggests that MitoQ supports both systolic function and reduces diastolic dysfunction in the aftermath of MI (Figure 2C,D).

The indices of myocardial contractility, represented by the maximum rates of pressure development (+dP/dt_max) and relaxation (−dP/dt_min), were significantly compromised in the MI group (Figure 2E,F). These reductions indicate impaired contractile and relaxation capabilities in MI‐affected hearts. MitoQ treatment, however, preserved only the maximum rate of relaxation (Figure 2F), highlighting MitoQ's effectiveness in maintaining myocardial relaxation after infarction.

When analyzing isolated papillary muscle function, the MI group displayed reduced force development (Figure 2G) as well as decreased rates of contraction (+dF/dt) (Figure 2H) and relaxation (−dF/dt) (Figure 2I), underscoring a decline in both contractile strength and kinetics. MitoQ treatment maintained developed force and contractile and relaxation velocities close to normal levels, indicating that MitoQ helps sustain contractile performance in the papillary muscles under post‐infarction conditions (Figure 2G–I).

Further, in the post‐rest potentiation analysis, MI animals showed a marked decline in force generation at 15, 30, and 60 s of rest (Figure 2J), suggesting reduced calcium reuptake or handling during these intervals. MitoQ treatment did not improve this reduction, although a tendency to improve in force generation was seen at 60 s (*p* = 0.08).

The calcium response curve revealed that MI animals exhibited diminished force generation across increasing calcium concentrations (0.62–3.75 mM), reflecting impaired calcium sensitivity or handling (Figure 2K). In contrast, MitoQ treatment enhanced the force response at each calcium level, suggesting improved calcium responsiveness and contractility (Figure 2K).

Finally, under β‐adrenergic stimulation with isoproterenol, MI animals did not display an increase in force response with increased isoproterenol concentration (Figure 2L) when compared to Sham or Sham treated with MitoQ. MitoQ treatment improved the force response to isoproterenol in MI (Figure 2L, *p* < 0.05 vs. 10^−10^), indicating enhanced β‐adrenergic responsiveness and potentially reduced β‐receptor desensitization.

### 3.3 Isolated Cardiomyocyte

**FIGURE 3:**
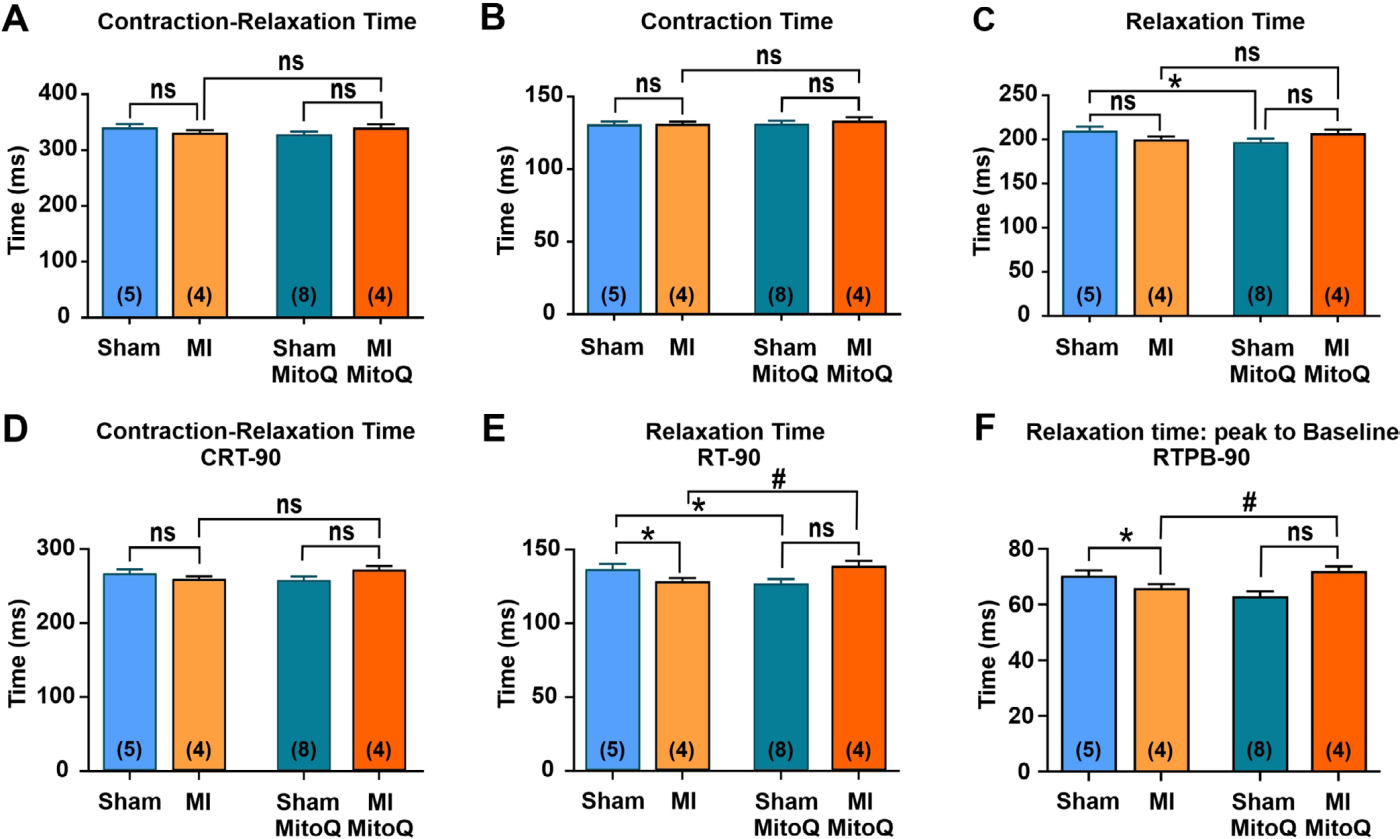
Effects of MitoQ on contraction and relaxation kinetics in isolated cardiomyocytes. (A) Overall contraction‐relaxation time (CRT); (B) contraction time (CT); (C) relaxation time (RT); (D) contraction‐relaxation time to 90% relaxation (CRT‐90). (E) time required to reach 90% relaxation (RT‐T90); (F) time from peak contraction to 90% of baseline (RTPB‐T90). Data are presented as mean ± SEM. Statistical analysis was performed using two‐way ANOVA. *p < 0.05 vs. Sham; #*p* < 0.05 vs. MI; ns, not significant. The number of animals per group is indicated in parentheses.

The data presented in Figure 3 describe the effects of MitoQ on the contraction and relaxation timing parameters in cardiomyocytes following myocardial infarction (MI). Panel 3A shows the overall contraction‐relaxation time (CRT), which was similar between groups, indicating that MI and MitoQ treatment did not affect the total duration of the contraction‐relaxation cycle. Panel 3B reveals that contraction time (CT) also remained unchanged between groups, suggesting no impact of MI or MitoQ on the duration of contraction specifically.

In contrast, Panel 3C illustrates that MitoQ significantly reduced the relaxation time (RT) in Sham animals when compared to the sham group (*p* < 0.05). Panel D examines the contraction‐relaxation time to 90% relaxation (CRT‐90), showing a significant increase in the MI group treated with MitoQ when compared to MI.

Panel E focuses on the time required to reach 90% relaxation (RT‐T90), which was shortened in the MI group and in the Sham treated with MitoQ, demonstrating faster relaxation kinetics. MitoQ treatment increased RT‐T90 in the MI.

Lastly, Panel F shows the relaxation time from peak contraction to 90% of baseline (RTPB‐T90). This parameter was significantly reduced in the MI group, and MitoQ treatment restored it closer to the sham group's values, further supporting MitoQ's positive effect on relaxation efficiency.

**FIGURE 4:**
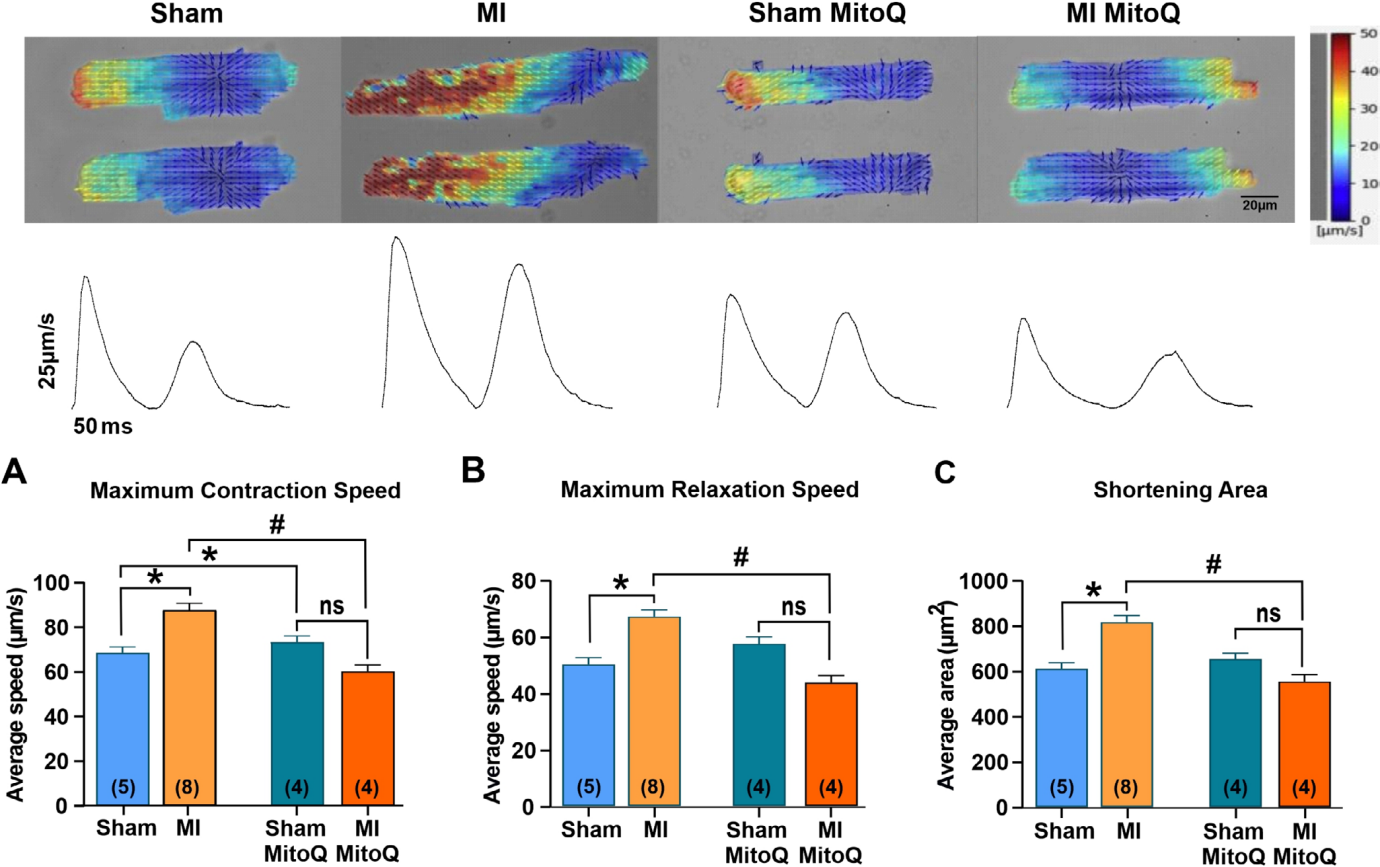
Evaluation of the effects of 7 days of treatment with MitoQ on the contractile mechanics of isolated cardiomyocytes. (A) Maximum contraction speed (μm/s); (B) maximum relaxation speed (μm/s); (C) shortening area (μm^2^). Data are presented as mean ± SEM. Statistical analysis was performed using two‐way ANOVA. *p < 0.05 vs. Sham; #*p* < 0.05 vs. MI; ns not significant. The number of animals per group is indicated in parentheses.

The results in Figure 4 highlight the effects of MitoQ on the contractile dynamics of cardiomyocytes after myocardial infarction (MI). Representative images of cardiomyocytes with velocity vector overlays illustrate changes in contraction and relaxation across groups, showing that MI increases the contractile speed, which is visually evident by the increased velocity patterns in untreated MI cells compared to sham and MitoQ‐treated groups.

In quantitative terms, Figure 4A shows that the maximum contraction speed was significantly higher in the MI group compared to sham (*p* < 0.05), indicating an increased contractile response post‐infarction. MitoQ treatment, however, reduced this contraction speed, bringing it closer to sham values, suggesting that MitoQ mitigates the exaggerated contractile response induced by MI (Figure 4A).

Figure 4B presents the maximum relaxation speed, which was also elevated in the MI group relative to sham controls (*p* < 0.05). MitoQ treatment prevented this increase, reducing the relaxation speed to levels comparable to the sham group. This result indicates that MitoQ aids in normalizing relaxation dynamics, countering the acceleration seen in MI‐affected cells.

Figure 4C shows the shortening area, a measure of the total contraction extent in cardiomyocytes. The MI group exhibited a significant increase in shortening area compared to sham, reflecting an abnormal extent of cellular contraction. MitoQ treatment reduced the shortening area in MI cells (*p* < 0.05), suggesting its role in preventing excessive contractile activity.

**FIGURE 5:**
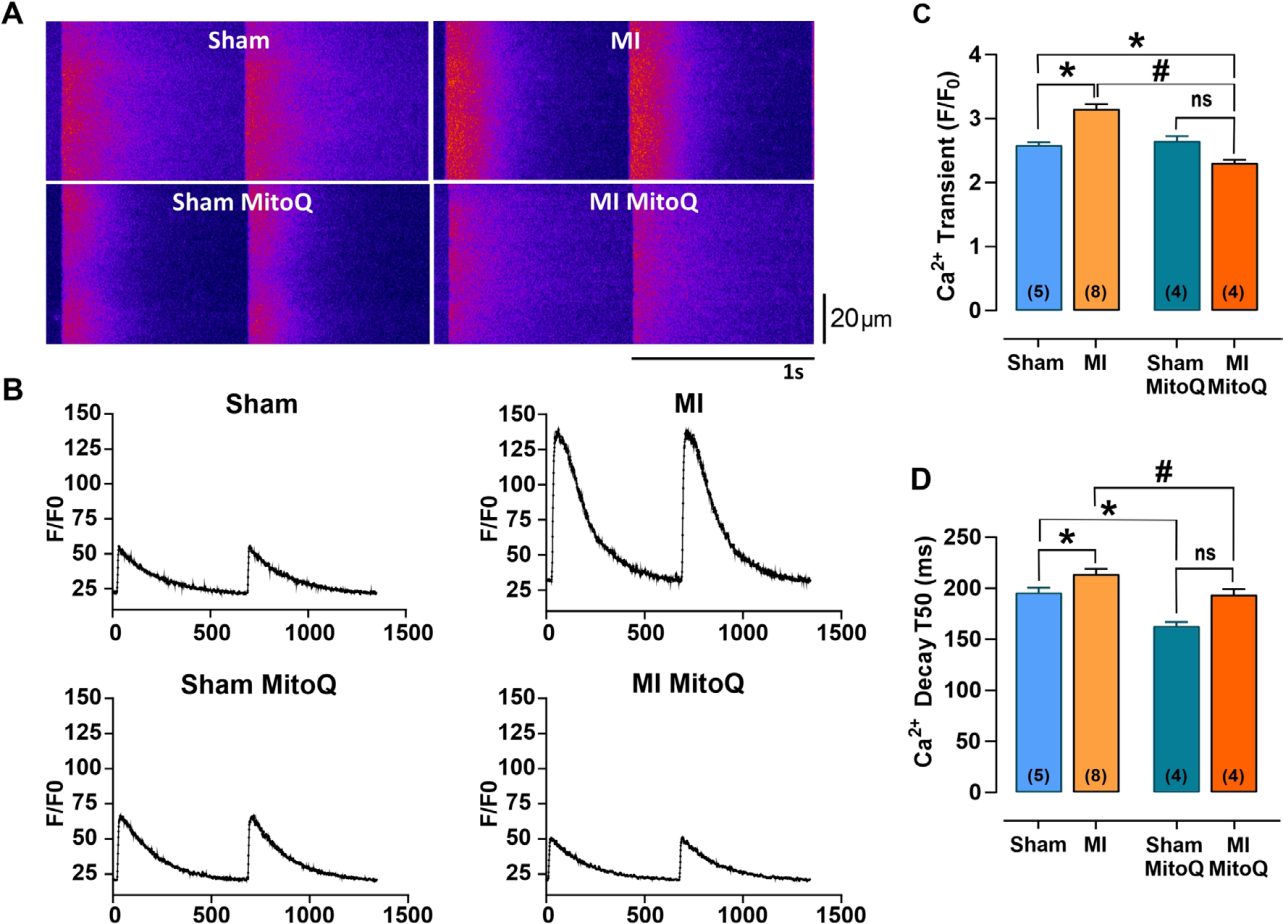
Treatment with MitoQ prevents contractile changes in isolated cardiomyocytes from rats subjected to 7 days post‐infarction. (A) Representative images of Ca^2+^ transient fluorescence, Scale = 20 μm; (B) representative trace of Ca^2+^ transient; (C) amplitude of Ca^2+^ transient; (D) time to 50% decay of Ca^2+^ transient. Approximately 20 cells per animal were analyzed. Data are presented as mean ± SEM. Statistical analysis was performed using two‐way ANOVA. *p < 0.05 vs. Sham; #*p* < 0.05 vs. MI; ns, not significant. The number of animals per group is indicated in parentheses.

The results in Figure 5 highlight the impact of MitoQ on calcium dynamics in cardiomyocytes following myocardial infarction (MI). In Panel A, representative fluorescence images of Ca^2+^ transients show differences in calcium signaling between the groups. The MI group exhibits increased Ca^2+^ transient intensity compared to the sham group (Figure 5C), indicating heightened calcium transient post‐infarction. MitoQ treatment in MI animals reduced this elevated calcium transient, suggesting a normalization effect on calcium signaling (Figure 5C).

Panel B shows representative Ca^2+^ transient traces, where MI results in a marked increase in transient amplitude, reflective of increased calcium handling. Panel C quantifies this effect, with the MI group displaying a significantly higher Ca^2+^ transient amplitude compared to sham (*p* < 0.05). MitoQ treatment in MI animals reduced the transient amplitude to levels closer to those of the sham group (*p* < 0.05 vs. MI), indicating MitoQ's effectiveness in modulating calcium amplitude post‐MI.

Panel D examines the Ca^2+^ decay time (T50), a parameter of calcium reuptake efficiency. The MI group shows prolonged T50, demonstrating slower calcium reuptake, which is typical of impaired cardiac relaxation. MitoQ treatment significantly reduced T50 in MI animals (*p* < 0.05 vs. MI), bringing it closer to sham values, indicating improved calcium reuptake kinetics and suggesting enhanced relaxation capacity.

**FIGURE 6:**
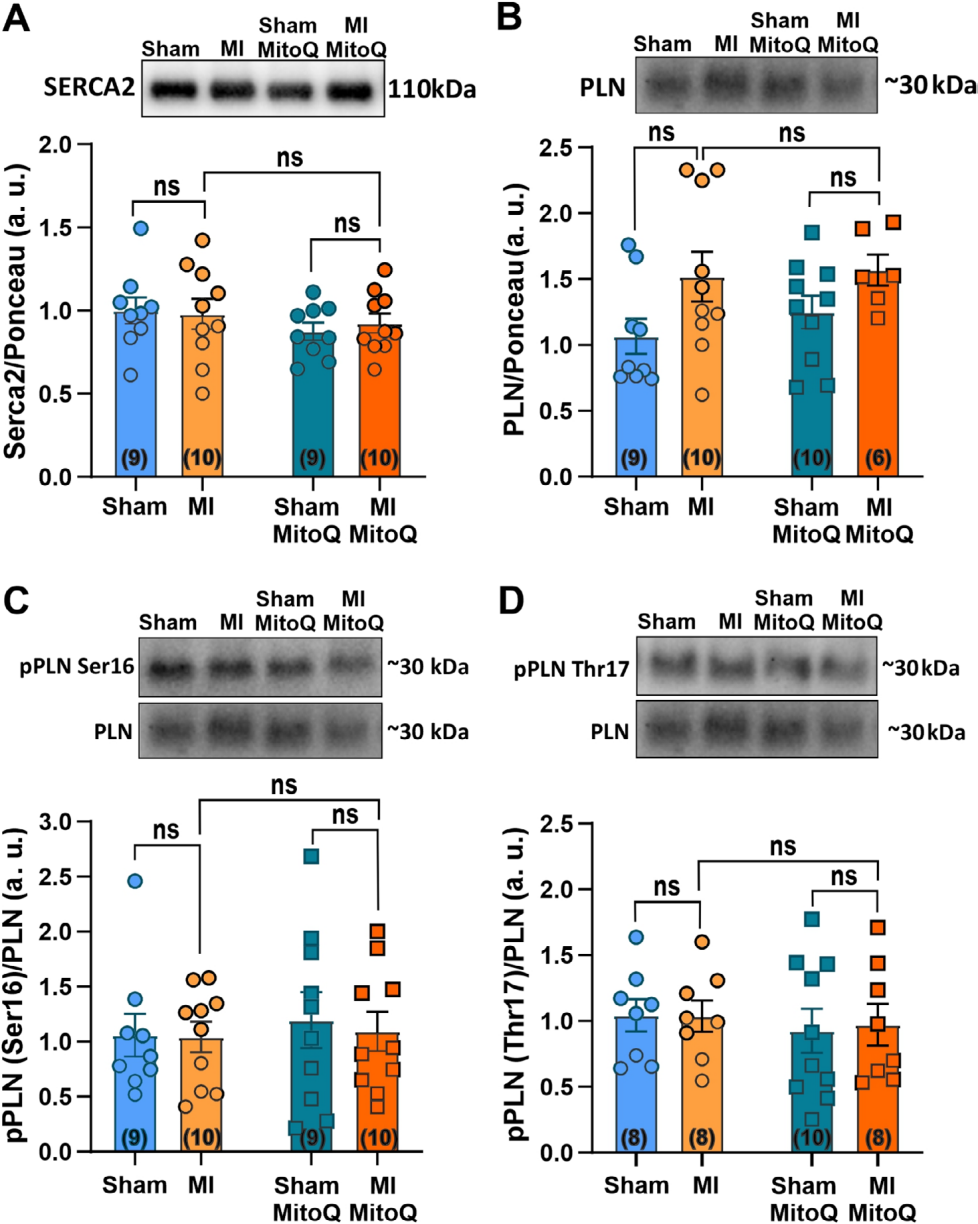
Effects of MitoQ treatment on the expression of proteins related to calcium handling in the myocardium post‐myocardial infarction (MI). (A) Western blot for sarcoplasmic reticulum Ca2 + ‐ATPase (SERCA2a) protein expression in left ventricular tissue; (B) Western blot for phospholamban (PLN) protein expression in left ventricular tissue; (C) phosphorylation of PLN at serine 16 (pPLN Ser16); (D) phosphorylation of PLN at threonine 17 (pPLN Thr17). Protein expression was normalized to total protein. Representative images are shown above for all analyses. Data are presented as mean ± SEM. Statistical analysis was performed using two‐way ANOVA. Differences were considered significant at *p* < 0.05. The number of animals per group is indicated in parentheses.

The results in Figure 6 demonstrate the effects of MitoQ treatment on the expression of proteins related to calcium handling in the myocardium post‐myocardial infarction (MI). In Panel A, the expression of sarcoplasmic reticulum Ca^2+^‐ATPase (SERCA2a) was assessed, showing no significant difference in expression levels among the sham, MI, sham MitoQ, and MI MitoQ groups. This indicates that MI and MitoQ treatment did not significantly alter SERCA2a expression.

Panel B shows phospholamban (PLN) expression, another key regulator of calcium cycling. Similar to SERCA2a, PLN levels were not significantly different across the experimental groups, suggesting that MI and MitoQ treatment did not affect PLN expression levels.

In Panel C, the phosphorylation state of PLN at serine 16 (pPLN Ser16) was examined. The results indicate that MI did not significantly change pPLN Ser16 levels compared to the sham group. MitoQ treatment in MI animals did not change phosphorylation at Ser16, suggesting no modulatory effect of MitoQ on PLN phosphorylation.

Finally, Panel D presents the phosphorylation status of PLN at threonine 17 (pPLN Thr17). Similar to pPLN Ser16, pPLN Thr17 levels were relatively consistent across the groups, with no significant differences detected. This consistency suggests that MI and MitoQ treatment did not markedly alter the phosphorylation of PLN at this site.

### 3.4 Mitochondrial ROS Production

**FIGURE 7:**
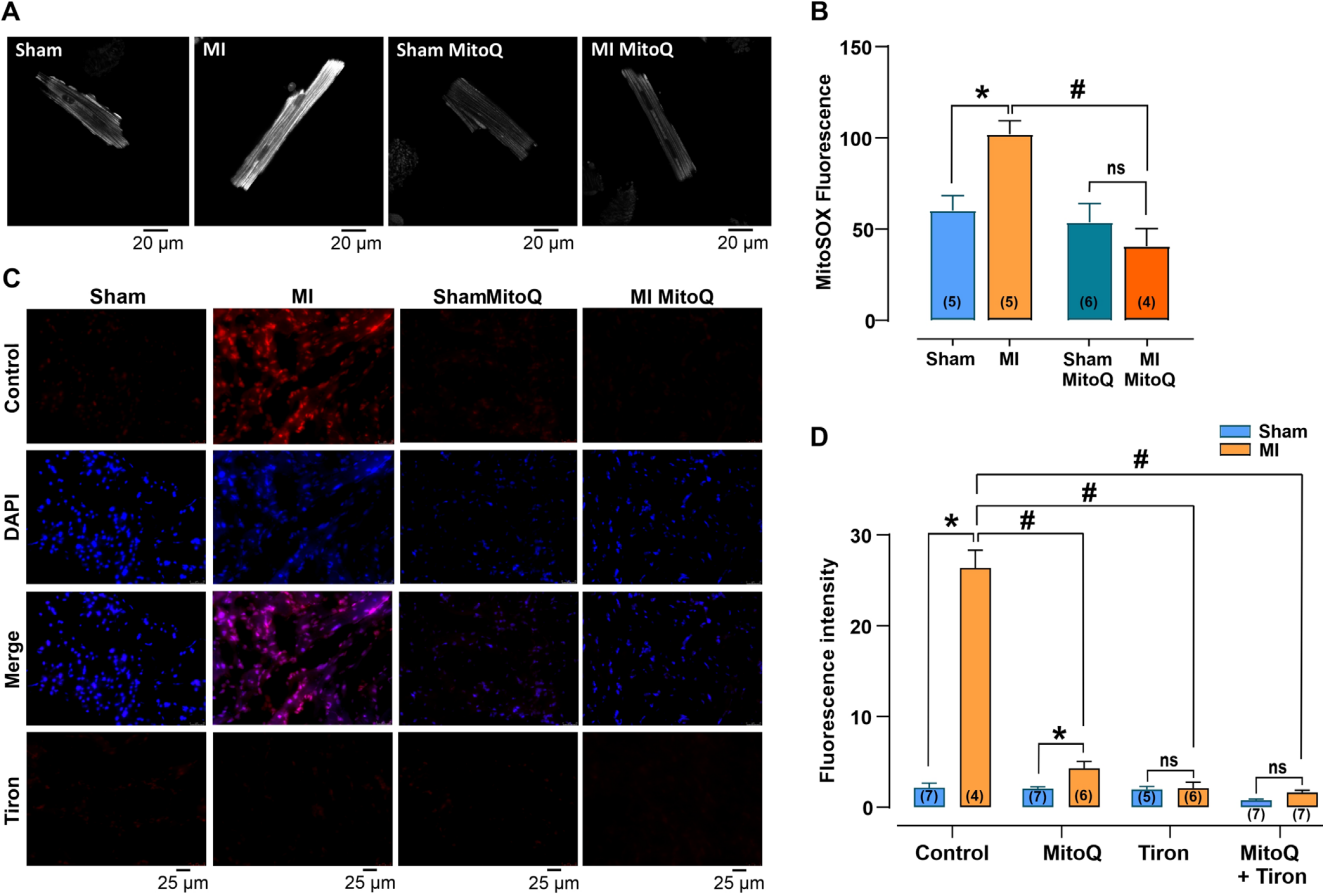
Effects of MitoQ treatment on mitochondrial superoxide (O_2_^•−^) production in cardiomyocytes after myocardial infarction (MI). (A) Representative images stained with MitoSOX, a fluorescent indicator for mitochondrial superoxide. Scale = 20 μm; (B) quantitative analysis of MitoSOX fluorescence; (C) representative images of myocardial tissue sections stained with dihydroethidium (DHE), a fluorescent probe used to assess reactive oxygen species (ROS) production. Scale = 25 μm. (D) Quantitative analysis of DHE fluorescence intensity. Data are presented as mean ± SEM. Statistical analysis was performed using two‐way ANOVA. *p < 0.05 vs. Sham; #*p* < 0.05 vs. MI. The number of animals per group is indicated in parentheses.

The results in Figure 7A,B illustrate the effects of MitoQ treatment on mitochondrial superoxide (O_2_^•−^) production in cardiomyocytes after myocardial infarction (MI). In Panel A, representative images stained with MitoSOX, a fluorescent indicator for mitochondrial superoxide, show increased fluorescence intensity in the MI group compared to the sham group, indicating elevated oxidative stress in the mitochondria post‐infarction. Quantitative analysis in Panel B confirms these findings, with the MI group exhibiting significantly higher MitoSOX fluorescence than the sham group (*p* < 0.05). MitoQ treatment in MI animals effectively decreased fluorescence to levels closer to the sham group (*p* < 0.05 vs. MI), supporting MitoQ's role in reducing oxidative stress in post‐MI cardiomyocytes.

### 3.5 Cellular ROS Production

Figure 7C provides additional insights by presenting images of myocardial tissue sections labeled with DHE (dihydroethidium), another indicator of ROS production. The MI group displayed an intense DHE fluorescence signal, which indicates increased ROS levels in the myocardial tissue. MitoQ treatment markedly reduced this signal in the MI group, further supporting its antioxidant properties.

Panel D quantifies DHE fluorescence intensity, showing that MI led to a significant increase in superoxide levels in the myocardium compared to sham (*p* < 0.05). MitoQ treatment significantly reduced fluorescence intensity in the MI group, bringing ROS levels closer to sham values (*p* < 0.05). Although MI animals treated with MitoQ still showed remaining ROS levels compared to sham (Figure 7D). The combination of MitoQ and Tiron resulted in additional reduction beyond either treatment alone, suggesting that MitoQ alone is not completely effective in managing oxidative stress in this context. These results indicate another source of ROS post‐MI.

**FIGURE 8:**
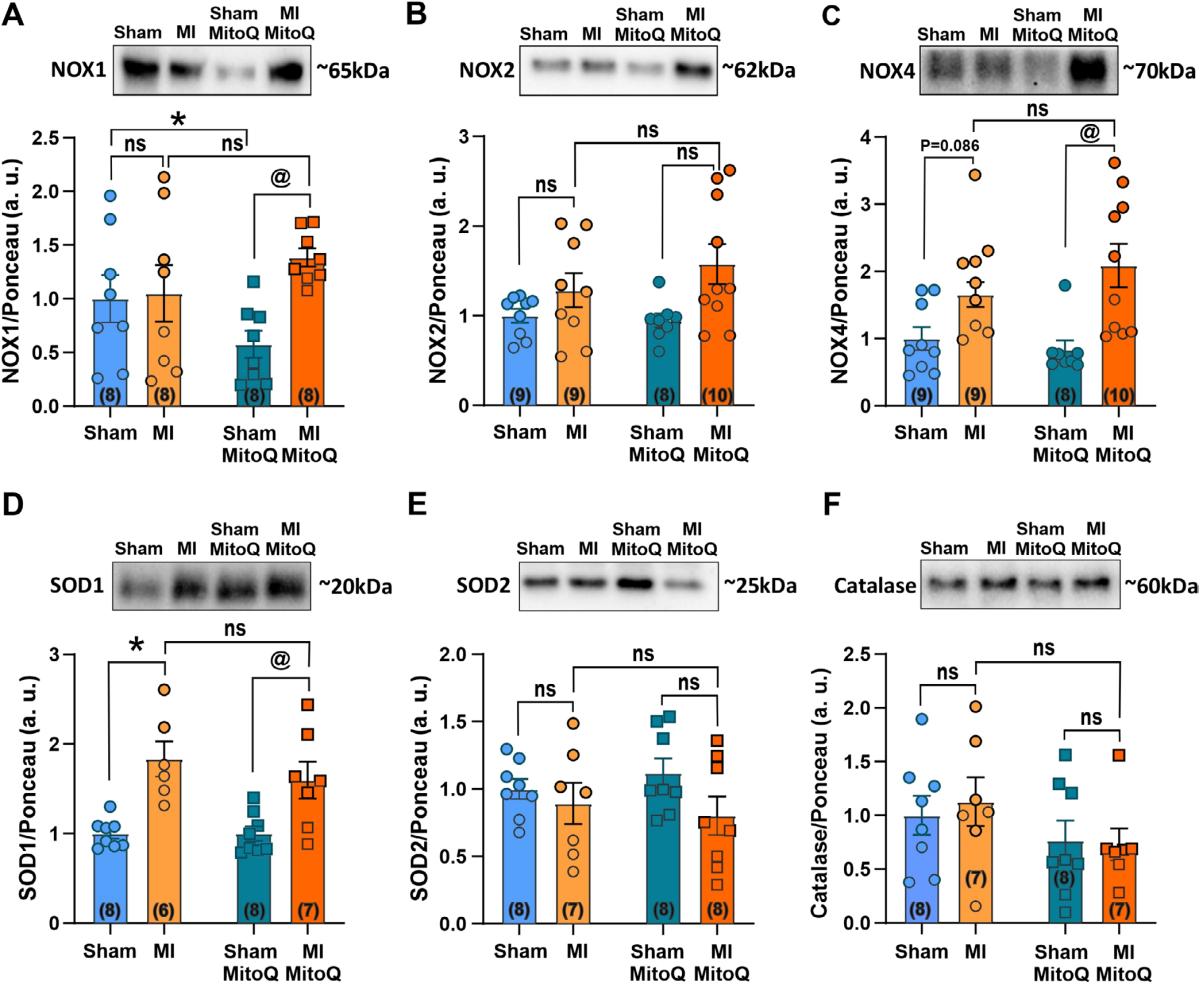
Effects of MitoQ on the expression of enzymes related to oxidative stress in myocardial tissue following myocardial infarction (MI). (A) Western blot for NOX‐1 protein expression in left ventricular tissue; (B) Western blot for NOX‐2 protein expression in left ventricular tissue; (C) Western blot for NOX‐4 protein expression in left ventricular tissue; (D) Western blot for SOD‐1 protein expression in left ventricular tissue; (E) Western blot for SOD‐2 protein expression in left ventricular tissue; (F) Western blot for Catalase protein expression in left ventricular tissue. Protein expression was normalized to total protein. Representative images are shown above for all analyses. Data are presented as mean ± SEM. *p < 0.05 vs. Sham. Statistical analysis was performed using two‐way ANOVA. Differences were considered significant at *p* < 0.05. The number of animals per group is indicated in parentheses.

The results in Figure 8 show the effects of MitoQ on the expression of enzymes related to oxidative stress in myocardial tissue following myocardial infarction (MI). Figure 8A shows that NOX1 expression is not increased in the MI group; instead, a reduction in NOX1 expression is observed only in the sham group treated with MitoQ, suggesting that MitoQ may selectively reduce NOX1 levels under non‐infarct conditions.

Figure 8B demonstrates that NOX2 expression remains stable between groups, with no elevation in the MI group and no change due to MitoQ treatment, indicating that NOX2 is unaffected by both MI and MitoQ intervention.

Figure 8C displays the expression of NOX4, another isoform of NADPH oxidase associated with ROS production. Similar to NOX2, NOX4 levels did not show significant changes across groups, suggesting that NOX4 is less responsive to MI or MitoQ treatment in this context.

SOD1 expression is significantly increased in the MI group compared to sham, indicating an upregulation in response to oxidative stress post‐infarction (Figure 8D). However, MitoQ treatment does not alter SOD1 levels in any group, suggesting that this antioxidant enzyme's response to MI remains unaffected by MitoQ (Figure 8D). Similarly, SOD2 expression did not change between groups, showing no significant differences due to MI or MitoQ treatment (Figure 8E). Catalase levels are also unchanged in all groups, indicating that this antioxidant enzyme is not impacted by MI or MitoQ (Figure 8F).

In summary, these findings suggest that MitoQ selectively reduces NOX1 expression in sham conditions but does not significantly affect the expression of NOX2, SOD1, SOD2, or catalase in the context of MI. This indicates that MitoQ's impact on oxidative stress‐related enzymes may be limited to specific proteins under particular conditions.

## 4 Discussion

This study provides robust evidence that the mitochondrial‐targeted antioxidant MitoQ mitigates systemic and cellular alterations during the early phase of myocardial infarction (MI) in a rat model. Untreated MI animals exhibited significant reductions in body weight gain, a hallmark of the catabolic state often associated with cardiac injury [23]. MitoQ treatment effectively attenuated this response, resulting in improved weight gain and suggesting a protective effect against MI‐induced metabolic derangements. This aligns with findings by Shimada [24], who also reported MitoQ's role in preserving metabolic balance in cardiac injury contexts.

Hypertrophic remodeling, a critical feature of post‐MI cardiac adaptation [19, 25, 26]. Remarkably, MitoQ normalized this parameter, demonstrating its potential to prevent maladaptive hypertrophy. These findings are consistent with prior evidence of MitoQ reducing hypertrophy in pressure‐overload models [17] and extend the observations of [18], who reported similar effects 4 weeks post‐MI. This suggests that MitoQ's anti‐hypertrophic effects are robust and persist across various cardiac stressors.

At the cellular level, MI induced significant morphological changes in cardiomyocytes, including increased cell area and length, indicative of hypertrophic remodeling. MitoQ treatment preserved cardiomyocyte dimensions, preventing these structural changes and maintaining cell morphology closer to sham controls. Interestingly, despite significant fibrosis observed in the MI group, MitoQ did not reduce collagen deposition during the acute phase. This suggests that MitoQ's protective effects are mediated through mechanisms other than direct anti‐fibrotic activity in the early stages of cardiac remodeling. As [18] demonstrated, mitochondrial ROS play a pivotal role in both early and late hypertrophic remodeling post‐MI, potentially linking ROS management to delayed anti‐fibrotic effects.

Beyond hypertrophy, MI animals exhibited elevated lung‐to‐body weight ratios, a clear indicator of pulmonary congestion caused by cardiac dysfunction [27]. Remarkably, MitoQ significantly reduced pulmonary congestion, suggesting an improvement in left ventricular function that mitigated the secondary rise in pulmonary pressure. This systemic benefit is further supported by hemodynamic data, which demonstrate that MitoQ effectively restored systolic and diastolic blood pressures to near‐normal levels. Furthermore, MitoQ preserved left ventricular systolic pressure (LVSP) and reduced left ventricular end‐diastolic pressure (LVEDP), indicating its role in maintaining both systolic function and mitigating diastolic dysfunction. These findings underscore MitoQ's broader systemic effects in mitigating complications associated with impaired cardiac output, highlighting its potential to alleviate pulmonary and circulatory burdens resulting from myocardial infarction.

Although infarct size was not significantly different among groups post‐myocardial infarction, our findings of improved cardiac function with MitoQ are consistent with the concept that functional recovery is not solely determined by infarct size, but also by the preservation of viable myocardium and attenuation of adverse remodeling.

Importantly, previous studies have demonstrated that early time points following myocardial infarction may not fully reflect structural remodeling processes, which evolve over time. Thus, the absence of differences in infarct size at 7 days does not preclude potential effects at later stages, such as 30 days, when scar maturation and remodeling are more established [17, 28].

In addition, alterations in the infarct border zone—including fibrosis, myofibroblast activity, and extracellular matrix remodeling—are known to significantly influence cardiac function independently of infarct size [17]. Furthermore, MitoQ has been shown to reduce oxidative stress and improve mitochondrial function, which may preferentially preserve viable myocardium in peri‐infarct regions [28].

Both NADPH oxidase and mitochondria are critical sources of reactive oxygen species in cardiomyocytes [3, 5, 7, 16, 29, 30].

The literature identifies NADPH oxidase, particularly its NOX2 and NOX4 isoforms, as key contributors to pathological cardiac remodeling by mediating hypertrophy and contractile dysfunction through redox‐sensitive signaling pathways [3, 5, 30].

NADPH oxidase activation is driven by humoral factors released after MI, including angiotensin II and aldosterone, which establish a feedback loop where ROS production from NADPH oxidase induces mitochondrial dysfunction, perpetuating a positive‐feedback cycle between NADPH oxidase and mitochondria [4, 14, 15, 31].

In the context of our findings, the preservation of cardiac function observed with MitoQ treatment can be interpreted as primarily resulting from its ability to reduce mitochondrial ROS. To quantify ROS production, we used two markers: cardiomyocyte mitochondria were labeled with MitoSOX, a mitochondrial‐specific marker, and cardiac tissue was labeled with DHE, a non‐specific marker for ROS. In isolated cardiomyocytes, MitoQ fully normalized mitochondrial ROS production in cells from MI animals, bringing levels down to those observed in sham controls. However, when assessing ROS in cardiac tissue using the non‐specific DHE marker, MitoQ did not completely normalize ROS production. A residual increase in ROS was still detected, approximately twice the basal level observed in sham animals. It is important to note that, due to the use of whole cardiac tissue for DHE labeling, it is not possible to determine whether this residual ROS originates from cardiomyocytes or from other cellular sources within the myocardium. In this context, inflammatory cells such as macrophages and activated fibroblasts are well‐established contributors to ROS production in the infarcted and peri‐infarct regions [17, 28].

However, this study did not directly assess the cellular origin of ROS through co‐localization approaches, which represents a limitation. Therefore, the contribution of specific cell populations cannot be definitively determined. Future studies using immunofluorescence co‐localization with cell‐specific markers (e.g., CD68 and α‐SMA) will be important to clarify the sources of oxidative stress in this setting.

Mitochondrial ROS and calcium signaling are intricately linked in maintaining cardiac function, particularly under stress conditions [3, 6, 13, 32]. In this study, increased calcium transients observed in isolated cardiomyocytes from MI animals reflect disruptions in calcium homeostasis driven by oxidative stress. Elevated mitochondrial ROS levels can oxidize key calcium‐handling proteins, including ryanodine receptors (RyR2) and SERCA2a [13], leading to calcium leakage from the sarcoplasmic reticulum (SR) and cytosolic calcium overload [8]. This dysregulation amplifies oxidative stress in a feed‐forward loop, further exacerbating calcium‐handling dysfunction [6, 13, 32].

The prolonged calcium decay time (Ca^2+^ Decay T50) observed in MI animals provides additional evidence for impaired SERCA2a activity [33], likely due to oxidative modifications [10]. Reduced SERCA2a function delays SR calcium reuptake, contributing to diastolic dysfunction, as evidenced by elevated left ventricular end‐diastolic pressure (LVEDP). Beyond mechanical dysfunction, prolonged cytosolic calcium exposure can promote mitochondrial calcium overload, increase ROS production and perpetuate redox imbalances [34].

MitoQ treatment normalized Ca^2+^ Decay T50 and reduced calcium transients, suggesting that attenuation of mitochondrial ROS mitigates oxidative damage to calcium‐handling proteins. Restoration of SERCA2a activity likely improves SR calcium reuptake and breaks the pathological cycle of redox signaling and calcium dysregulation. Importantly, the reduction in calcium transients highlights improved coordination between the SR and mitochondria, emphasizing MitoQ's role in stabilizing calcium‐redox signaling in this microdomain [10, 13].

These findings align with previous studies demonstrating that mitochondrial ROS disrupt calcium‐handling machinery by oxidizing RyR2 and impairing SERCA2a activity [9, 10, 35, 36, 37, 38, 39]. Furthermore, evidence from ischemia–reperfusion models indicates that SERCA overexpression improves mitochondrial quality control, underscoring the interplay between mitochondrial function, calcium regulation, and redox homeostasis [40]. Together, these data suggest that targeting mitochondrial ROS with MitoQ alleviates oxidative stress‐induced calcium dysregulation and preserves calcium‐handling efficiency.

This study highlights that normalizing mitochondrial ROS production plays a central role in improving myocardial contractility, correcting calcium mishandling, and alleviating diastolic dysfunction in the context of myocardial infarction. By reducing oxidative stress, the restoration of mitochondrial and sarcoplasmic reticulum calcium homeostasis is achieved, as evidenced by improved calcium transient dynamics. These adjustments break the pathological feedback loop between oxidative stress and calcium dysregulation, mitigating key contributors to myocardial remodeling and functional decline.

Clinically, integrating mitochondrial ROS normalization strategies into existing heart failure treatments could pave the way for more effective management of myocardial dysfunction and remodeling across various cardiac pathologies. Current therapies for heart failure or myocardial infarction primarily target humoral mechanisms, such as the renin‐angiotensin‐aldosterone system or sympathetic nervous system, but fail to directly address intracellular pathways involved in disease progression. Notably, there are no effective treatments currently available that specifically target intracellular mechanisms, such as redox imbalances or calcium mishandling, which are critical drivers of myocardial remodeling and dysfunction. By addressing these intracellular pathways, mitochondrial ROS normalization could fill a crucial gap in therapeutic approaches, offering a novel and complementary strategy for improving cardiac outcomes.

## Author Contributions

**Carolina Falcão Ximenes:** investigation, writing – original draft, writing – review and editing, conceptualization, visualization, data curation, formal analysis, methodology. **Marlon Ramos Rosado Machado:** investigation. **Carmen Castardeli:** investigation, methodology. **Pietra Zava Lorencini:** investigation, methodology, data curation, formal analysis. **Katyana Kaline Silva Ferreira:** investigation, visualization, data curation, methodology, formal analysis. **Sérgio Ricardo Aluotto Scalzo:** investigation, methodology, software, data curation, formal analysis. **Marcos Eliezeck:** formal analysis, methodology, investigation, data curation. **Silvia Guatimosim:** methodology, visualization, conceptualization, investigation, data curation. **Anna Karolina Nascimento Costa:** investigation, methodology. **Eduardo Hertel Ribeiro:** writing – original draft, visualization, methodology, data curation, conceptualization, formal analysis. **Sara Bianca Oliveira Mendes:** investigation, methodology. **Ivanita Stefanon:** data curation, supervision, formal analysis, project administration, resources, writing – review and editing, writing – original draft, funding acquisition, conceptualization, methodology. **Mario Morais Silva:** investigation, visualization, data curation, methodology, formal analysis. **Aurélia Araujo Fernandes:** investigation, methodology, formal analysis, data curation. **Donald M. Bers:** investigation, methodology, data curation, formal analysis.

## Funding

This work was funded by grant TO2022‐6C3F7, Edital 019/2022, Espirito Santo Foundation for Research and Innovation Support (FAPES) and protocol 151271/2024‐0 CNPq‐Brazil; Fundação de Amparo à Pesquisa do Estado de Minas Gerais (FAPEMIG): APQ‐04203‐23 and APQ‐02501‐25; CNPq 442473/2023‐0, 407654/2025‐9 and 444251/2024‐3; FAPEMIG APQ‐04900‐22.

## Conflicts of Interest

The authors declare no conflicts of interest.

## Data Availability

The data supporting the findings of this study are available in the doctoral thesis deposited in the CAPES Sucupira Platform (Brazil) and from the corresponding author upon reasonable request.
